# How the Camel’s Back Breaks: A Scoping Review of Cumulative Occupational Distress and the Conceptual Absence of Micro-Trauma in Nursing

**DOI:** 10.3390/nursrep16090299

**Published:** 2026-08-25

**Authors:** Megan Dale, Maude Chapman, Caryn West

**Affiliations:** 1 Nursing and Midwifery, College of Healthcare Sciences, James Cook University, Townsville, QLD 4810, Australia; maude.chapman@jcu.edu.au (M.C.); carwest@csu.edu.au (C.W.); 2 School of Nursing, Paramedicine and Healthcare Sciences, Charles Sturt University, Bathurst, NSW 2795, Australia

**Keywords:** burnout, compassion fatigue, cumulative trauma, occupational distress, micro-trauma, nurse, nursing, occupational trauma, occupational health, psychological stress, scoping review

## Abstract

**Background/Objectives:** Nursing involves repeated exposure to emotionally, morally, and psychologically distressing experiences, yet the mechanisms through which low-intensity occupational stressors accumulate and contribute to psychological harm remain conceptually unclear. This scoping review examined how the nursing literature conceptualises the cumulative effects of repeated occupationally distressing experiences and identified the conceptual gap created by the absence of a unifying term such as micro-trauma. **Methods:** A scoping review was undertaken to map concepts used to describe cumulative occupational distress in nursing and determine whether any terminology captures the cumulative impact of repeated low-intensity exposures. Searches were conducted across CINAHL, MEDLINE, and Emcare, supplemented by citation searching, Google Scholar, targeted grey literature searching and structured generative artificial intelligence-assisted source identification. Data were extracted using a priori criteria and synthesised narratively. Consistent with Joanna Briggs Institute guidance, quality appraisal was not undertaken. **Results:** No included publication applied the term ‘micro-trauma’ to nursing or healthcare workers. Seven publications met the inclusion criteria following expanded searching across supplemental pathways. The literature used heterogeneous and overlapping terminology, including burnout, compassion fatigue, cumulative grief, moral distress, workplace trauma, and post-traumatic stress disorder symptomatology, to describe cumulative psychological burden, but none provided a unifying conceptual frame for repeated low-intensity exposures. **Conclusions:** The current literature does not yet clearly define or consistently apply micro-trauma in nursing, despite evidence that repeated occupational exposures contribute to cumulative psychological burden. This review highlights a conceptual space before end-point conditions such as burnout, compassion fatigue, or post-traumatic stress disorder symptomatology are recognised. Future research should develop and test a clearer conceptual framework for micro-trauma in nursing before it is applied as a distinct unifying concept.

## 1. Introduction

Nursing work is characterised by repeated exposure to emotionally, morally, and psychologically distressing experiences that accumulate over time and shape nurses’ health and well-being [1,2]. The frequency and impact of occupational stressors, workplace violence, and trauma-related outcomes in nursing are well documented [2,3,4]. These exposures often occur at low intensity but high frequency, forming part of the routine fabric of clinical practice and contributing to cumulative psychological burden [5]. Understanding how these repeated exposures accumulate conceptually is essential for clarifying upstream mechanisms of harm before end-point conditions such as burnout or PTSD symptomology emerge.

Micro-trauma is described in occupational health and psychoanalytic literature as repeated low-intensity emotionally or morally distressing events that may not meet the threshold for trauma when considered in isolation, but may accumulate over time and contribute to psychological burden [6,7]. Although micro-trauma has been explored in other disciplines, its relevance to nursing has not been clearly articulated. In nursing, day-to-day experiences such as emotional labour, moral tension, interpersonal conflict, and exposure to suffering may accumulate, yet these experiences are often normalised, minimised, or absorbed into expectations of professional resilience [1,8,9,10].

Despite increasing recognition of distress in nurses, limited conceptual clarity exists on how low-intensity occupational exposures accumulate and contribute to cumulative psychological harm [1]. Theoretical models such as the job demands–resources (JD–R) model [11,12] and the transactional model of stress and coping [13,14] provide a foundation for understanding how repeated exposures interact with appraisal processes, resource availability, and coping demands to produce cumulative burden. However, these models do not offer terminology that captures the small, repeated experiences that precede end-point conditions.

A multitude of interchangeable terms are currently used to describe the physical, psychological, and emotional symptomology experienced by nurses, including compassion fatigue, cumulative grief, secondary traumatic stress, vicarious trauma, and moral distress [1]. These constructs largely describe manifestations or outcomes of occupational distress rather than the cumulative effects of repeated workplace exposures that precede them [5,15]. Consequently, the literature is characterised by conceptual fragmentation, with related phenomena described through multiple partially overlapping terms and theoretical perspectives.

Cumulative trauma is an emerging concept that encompasses frequent or repeated exposure to potentially distressing events, both direct and indirect [16]. Although cumulative trauma overlaps with micro-trauma, the constructs are not interchangeable. Preliminary searching indicated that micro-trauma is not used in nursing literature, highlighting a gap in how the profession describes repeated low-intensity occupational exposures before end-point conditions manifest.

## 2. The Review

### Aim

A scoping review was undertaken to examine how the nursing literature conceptualises the cumulative effects of repeated occupationally distressing experiences and to identify the conceptual gap created by the absence of a unifying term such as micro-trauma. The revised research question guiding the study was: What concepts are used in nursing literature to describe the cumulative effects of repeated occupationally distressing experiences on nurses’ health and well-being? To address this question, the review explored:

1. What terminology is used to describe repeated occupationally distressing experiences in nursing.
2. How included studies conceptualise cumulative processes arising from repeated low-intensity exposures.
3. What health and well-being outcomes are associated with cumulative occupational distress.
4. What conceptual gaps emerge from the absence of a unifying term such as micro-trauma.

## 3. Methods

### 3.1. Design

A scoping review methodology was selected to map how the nursing literature conceptualises repeated occupationally distressing experiences and to clarify the conceptual ambiguity concerning cumulative occupational distress. Preliminary searching indicated that the term “micro-trauma” is not used in nursing literature, and related trauma terminology is applied inconsistently and interchangeably. Because the literature lacked a unifying term, a scoping review was appropriate to map existing concepts rather than conduct a concept analysis. The review therefore examined cumulative trauma and related terminology as the closest conceptual descriptors of repeated low-intensity occupational exposures. The transactional model of stress and coping [13] and the job demands–resources model [11] provided a theoretical foundation for interpreting how repeated exposures accumulate over time and contribute to cumulative burden.

The review followed the Joanna Briggs Institute (JBI) nine-stage scoping review framework [17], including defining the objective and review question, developing eligibility criteria, planning and conducting the search, selecting and extracting evidence, analysing and presenting results, and summarising implications. Transparency and rigour were supported by conducting the review in accordance with JBI methodological guidance and reporting it using the Preferred Reporting Items for Systematic Reviews and Meta-Analyses Extension for Scoping Reviews (PRISMA-ScR) [18] (Supplementary Marterials, Table S1). A protocol was not registered due to the exploratory nature of the review and the evolving conceptual focus. Subsequent refinements, including the broadened healthcare worker search and supplementary AI-assisted source identification, are described in the search methods to support transparency.

### 3.2. Search Methods

An initial exploratory literature search was conducted in consultation with a senior health sciences librarian to identify relevant keywords, subject headings, and Boolean operators. Preliminary searching was undertaken in the Cumulative Index to Nursing and Allied Health Literature (CINAHL), Ovid MEDLINE, Scopus, and Ovid Emcare to identify relevant medical subject headings (MeSH). Structured exploratory queries were also run in Perplexity as part of AI-assisted source identification to identify additional terminology used in related fields. These terms informed the development of the formal search strategy. Consensus was reached that the selected terms were appropriate for capturing literature related to cumulative occupational distress in nursing.

Consistent with JBI scoping review guidance, the objective, review question, and eligibility criteria were aligned using population–concept–context logic [17]. The population was registered nurses and nurse-relevant healthcare populations, the concept was cumulative occupational distress and related terminology, and the context was occupational nursing and healthcare workers. The SPICE framework [19] was then used pragmatically to translate the review question into searchable concepts. SPICE supported structured searching for perspectives of registered nurses, the phenomenon of cumulative occupational distress, and the evaluation of health and well-being outcomes. Setting and comparison were not restricted to maximise conceptual capture across nursing and nurse-relevant healthcare contexts.

Formal searching was undertaken using multiple Boolean combinations in three databases (CINAHL, MEDLINE, and Emcare) between March and May 2024. No date limits were applied to ensure comprehensive coverage, as cumulative occupational distress has been described across multiple decades. The search was limited to English-language publications because translation resources were unavailable. Full-text access was sought through institutional subscriptions, open-access sources, publisher websites, and citation searching. Records for which full texts could not be retrieved after reasonable attempts were excluded at the screening stage, which may have introduced availability bias. Keywords were finalised using the SPICE framework [19] to reflect the revised review question, as shown in Table 1.

Following consultation with senior university librarians, the search protocol was expanded to include structured generative AI-assisted source identification. Perplexity, Microsoft Copilot, and Elicit were used to identify additional terminology and potentially relevant sources not retrieved through database searching. The use of AI-assisted source identification was informed by emerging guidance on AI-supported literature searching and review processes [20,21,22]. AI-assisted source identification was used only to identify candidate sources for verification and did not replace database searching, eligibility screening, or reviewer judgement.

Structured queries were developed to identify literature describing repeated occupationally distressing experiences in nurses, including terms such as ‘cumulative trauma’, ‘repeated exposure’, and ‘occupational distress in nurses’. Searches were conducted between March and May 2024 using standard settings available at that time. No paid versions or enhanced features were used. Because generative AI outputs are dynamic and may vary by tool version and retrieval environment, AI-assisted source identification was treated as a transparent supplementary source-identification strategy rather than a fully reproducible systematic search pathway.

Ethical considerations included transparency in AI use, avoidance of bias, and verification of all AI-identified sources. AI-generated records were screened using the same protocol as database-derived records, and inclusion criteria remained consistent across all search pathways.

Grey literature and Google Scholar searches were conducted using the same conceptual terms. The first three pages of Google Scholar results were screened, identifying six potentially relevant records for full-text retrieval. Targeted grey literature searching was limited to .gov and .org domains. Records were retained only where they addressed repeated occupational exposure, cumulative trauma, cumulative grief, workplace trauma, or nurse-relevant psychological trauma.

A three-step process was applied: (1) initial database searching and hand searching; (2) repeated searching using broadened healthcare worker terms; and (3) supplementary searching using generative AI tools, Google Scholar, and targeted grey literature. This staged approach aligns with PRISMA-ScR guidance for transparent reporting of information sources, search limits, and supplementary pathways.

### 3.3. Inclusion and Exclusion Criteria

The inclusion criteria comprised articles and selected grey literature reports that: (1) were published in English; (2) addressed registered nurses directly or healthcare populations in which nurses were explicitly included, discussed, or reasonably represented within the occupational context; and (3) examined repeated occupationally distressing experiences, cumulative exposure, cumulative psychological burden, or health and well-being outcomes associated with repeated occupational exposure. Sources addressing broader healthcare literature were retained only where they directly examined repeated occupational exposure, cumulative trauma, cumulative grief, workplace trauma, or related cumulative psychological burden relevant to nursing work. Where nurses were not analysed separately, but identified, findings were treated as indirect contextual evidence and were not interpreted as nurse-specific. Because explicit micro-trauma terminology was not identified in preliminary nursing searches, cumulative trauma-related terminology was operationalised as the closest conceptual descriptor of repeated low-intensity exposures.

Sources were excluded if they: (1) were not published in English; (2) did not address nursing or nurse-relevant healthcare populations; (3) focused solely on single acute traumatic events without repetition or accumulation; or (4) examined only clinical diagnosis, prevalence, or intervention outcomes without conceptual relevance to cumulative occupational distress.

For screening and synthesis, repeated exposure was defined as recurrent, prolonged, or repeated occupational contact with distressing events or conditions over time. Occupationally distressing experiences included workplace violence, verbal aggression, patient death and dying, errors or witnessed errors, resource constraints, emotional labour, and morally challenging care. End-point terms such as burnout, compassion fatigue, post-traumatic stress symptoms, secondary traumatic stress, vicarious trauma, reduced well-being, impaired professional functioning, and workforce attrition were retained only where they were linked to cumulative occupational distress. Records were managed manually, and no dedicated systematic review screening software was used. Search results were exported from each database, and AI-assisted, Google Scholar, and grey literature search outputs were recorded in a screening spreadsheet. Duplicates were identified and removed manually before title and abstract screening.

### 3.4. Search Outcome

The initial database search retrieved 1790 results (CINAHL *n* = 1007, MEDLINE *n* = 730, Emcare *n* = 53). After manual removal of duplicates, 1574 titles and abstracts were screened independently by MD and CW using the revised eligibility criteria focused on cumulative occupational distress. Conflicts were resolved through discussion, with MC available as a third reviewer. Reviewer agreement statistics were not calculated. Citation-list hand searching did not identify additional eligible records. No studies from this first search met the inclusion criteria, because retrieved records did not conceptualise repeated low-intensity exposures or cumulative occupational distress. Instead, they focused on end-point diagnoses, general occupational stress, or interventions without addressing repetition or accumulation.

The second search substituted the keyword ‘nurse’ with ‘healthcare worker’ and retrieved 17 records (CINAHL *n* = 4, MEDLINE *n* = 4, Emcare *n* = 9). No duplicates were identified. Titles and abstracts were screened independently by MD and CW, with disagreements resolved through discussion and by MC where required. No studies from this second search met the inclusion criteria, because records did not conceptualise repeated occupational exposure or cumulative processes in a way relevant to nurses or nurse-relevant healthcare populations.

The generative artificial intelligence source identification process elicited 27 results (Perplexity *n* = 14, Microsoft Copilot *n* = 5, Elicit *n* = 8). After manual removal of duplicates (*n* = 7), 20 records were screened using the same eligibility criteria as database-derived records. Fourteen records were excluded at title and abstract screening because they did not address nursing or nurse-relevant healthcare populations, did not examine repeated occupational exposure or cumulative processes, or focused solely on end-point diagnoses or interventions. Six records proceeded to full-text review, and two were excluded because they did not meet the conceptual relevance criteria. Four studies were identified as eligible through AI-assisted source identification.

Citation searching, hand searching, Google Scholar, and targeted grey literature searching identified a further three studies. Google Scholar screening of the first three results pages identified six potentially relevant records for full-text retrieval, and three met the inclusion criteria. Targeted grey literature searching (.gov and .org domains) identified one additional eligible record. The final seven included publications therefore comprised four records identified through AI-assisted source identification and three records identified through supplementary searching. No eligible publications were identified through the initial nursing-specific or broadened healthcare worker database searches.

**Figure 1:**
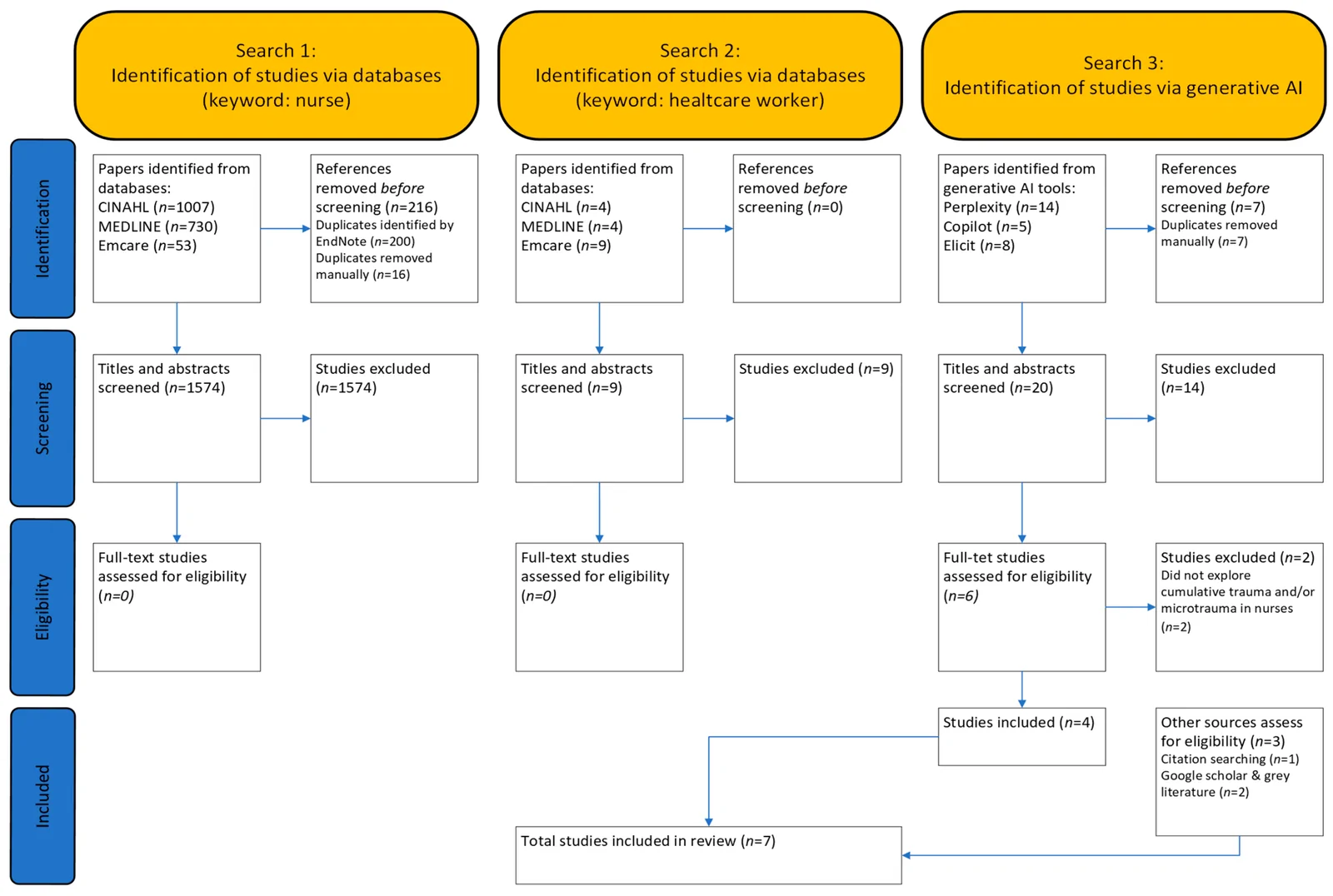
PRISMA flow diagram of study selection process.

Full-text screening of all potentially eligible articles was undertaken independently by MD and MC, with conflicts resolved by CW. Reasons for exclusion included lack of repeated exposure or accumulation, absence of a nursing or nurse-relevant healthcare population, focus on single acute traumatic events, and focus on diagnosis or intervention outcomes without conceptual relevance to cumulative occupational distress. Figure 1 presents the PRISMA-ScR flow chart of the article selection process [18]. Table 2 presents the source pathway for each included publication.

### 3.5. Data Abstraction and Synthesis

Guided by the JBI principles of data extraction [26], an extraction spreadsheet was developed, pilot-tested based on the a priori protocol, agreed on by all authors, and discussed throughout the extraction process. Extracted data included author/s, year published, country of origin, research aim, methodology, participants, key concepts, descriptions of repeated or cumulative exposure, and major relevant findings. Table 3 presents the data extraction findings chronologically to generate a timeline of the published literature and to illustrate how terminology describing cumulative occupational distress emerged across the included sources.

The extraction process was designed to chart and summarise the available evidence rather than to undertake formal concept analysis. Accordingly, the review mapped terminology, populations, evidence characteristics, cumulative exposure patterns, and reported outcomes, but did not attempt to generate defining attributes, model cases, antecedents, consequences, or empirical referents for any single concept. Due to the heterogeneity of the included studies, it was not possible or appropriate to conduct a meta-analysis. Findings were therefore synthesised descriptively and narratively. Narrative synthesis focused on how included studies described repeated occupational exposure, cumulative processes, and associated health and well-being outcomes.

### 3.6. Quality Appraisal

Consistent with JBI guidance, quality appraisal was not conducted, because the aim of this scoping review was to map the existing literature and identify conceptual gaps rather than assess methodological quality [26,27]. The absence of quality appraisal means included sources were not ranked by methodological strength. Instead, they were used to describe the scope of the evidence, terminology, and conceptual patterns related to repeated occupational exposure and cumulative occupational distress.

## 4. Results

### 4.1. Absence of Explicit Micro-Trauma Terminology in Nursing Literature

The principal finding was that the term ‘micro-trauma’ did not appear in any of the seven included publications. Instead, repeated occupationally distressing experiences were described using cumulative trauma, cumulative grief, workplace trauma, and nurse-specific psychological trauma. End-point terms, including burnout, compassion fatigue, moral distress, secondary traumatic stress, vicarious trauma, and post-traumatic stress symptoms, were present as outcomes or sequelae of repeated exposure, rather than as concepts describing cumulative processes. Cumulative trauma was therefore synthesised as the closest adjacent concept.

Table 4 summarises the cumulative trauma and cumulative exposure terminology identified across the included literature. Table 5 maps how each included publication addresses accumulation, repeated exposure, and associated end-point outcomes.

### 4.2. Characteristics of the Literature

The seven included articles were analysed for their relevance to repeated occupational exposure and cumulative occupational distress in nursing. Publication dates ranged from 1985 [23] to 2022, with six articles published between 2018 and 2022 [1,5,15,16,24,25]. Four articles originated from the United States of America, two from Australia, and one from the United Kingdom.

Four studies examined nursing as an all-inclusive group, one focused specifically on forensic mental health nurses, and one included both nurses and doctors working in an intensive care unit. Bywood and McMillan [16] was retained as indirect cross-occupational evidence because it examined repeated exposure to work-related trauma across non-frontline occupational groups, including healthcare workers, teachers, and social workers. Nurses are discussed as a healthcare occupation exposed to direct and indirect trauma, and nursing-related evidence is represented within the broader healthcare evidence base; however, nurses were not isolated, analysed, or reported as a distinct occupational subgroup. Therefore, the findings inform the broader cumulative work-related trauma literature and terminology, but do not constitute direct nurse-specific evidence.

Across the included literature, cumulative trauma and related terminology were used to describe repeated or prolonged occupational exposure and its effects on nurses’ psychological health, well-being, and professional functioning.

### 4.3. Descriptive Mapping of Included Evidence

#### 4.3.1. Concepts Used to Describe Cumulative Occupational Distress

The included literature used cumulative trauma and closely related cumulative exposure terminology to describe repeated occupational distress in nursing. Davidson and Jackson [23] applied cumulative trauma to explain long-term symptoms and maladaptive responses arising from repeated trauma-related experiences. Kapoor et al. [24] described cumulative grief associated with repeated exposure to death and dying in intensive care settings. Newman et al. [15] used workplace trauma to describe repeated exposure to violence, abuse, and threats to safety in forensic mental health nursing. Foli et al. [25] and Foli [5] developed nurse-specific psychological trauma terminology, including system-induced trauma, second-victim trauma, vicarious trauma, disaster trauma, and insufficient resource trauma. Boyle and Steinheiser [1] and Bywood and McMillan [16] discussed end-point outcomes such as burnout, compassion fatigue, moral distress, secondary traumatic stress, vicarious trauma, and post-traumatic stress symptoms. Collectively, the literature demonstrated recognition of cumulative occupational harm, although not through a single unifying term.

#### 4.3.2. Repeated Occupational Exposures

Across the included studies, cumulative occupational distress was linked to repeated exposure to emotionally, morally, physically, and/or organisationally distressing events. Repeated exposure to death, dying, and unresolved grief was described in intensive care contexts through cumulative grief and the use of ritualised acknowledgement such as the sacred pause [24]. Workplace aggression, repeated verbal abuse, physical violence, sexual violence, self-harm events, and threats to safety were identified in forensic mental health nursing as forms of workplace trauma that may accumulate over time [15]. Errors, witnessed errors, system-induced trauma, insufficient resource trauma, vicarious trauma, and second-victim experiences were described within nurse-specific psychological trauma literature [5,25]. Sustained emotional labour, moral distress, compassion fatigue, and organisational emotional hazards were also identified as routine features of nursing work that may contribute to cumulative burden [1]. Bywood and McMillan [16] provided indirect contextual evidence that repeated direct and indirect exposure to work-related trauma may contribute to cumulative psychological harm across emotionally demanding occupational groups, including healthcare workers, teachers, and social workers. Nurses are identified in the report as one healthcare group exposed to trauma; however, the review did not extract, analyse, or report findings of nurses separately. The source was therefore used to contextualise cumulative trauma terminology and cross-occupational relevance, not to support direct claims about nurses. This distinction is important because some exposures are overtly traumatic, while others are embedded within routine care and may be normalised until harm becomes visible through end-point terminology.

#### 4.3.3. Cumulative Processes

The included studies conceptualised cumulative processes as the mechanisms through which repeated exposures became layered over time. Davidson and Jackson [23] described cumulative trauma as the delayed effect of repeated trauma-related experiences. Kapoor et al. [24] framed repeated exposure to death and dying as cumulative grief and emphasised the value of ritualised acknowledgement. Newman et al. [15] described cumulative exposure to workplace violence and abuse as increasing vulnerability to psychological distress. Foli et al. [25] and Foli [5] explained nurses’ psychological trauma through cumulative burden and allostatic load. Boyle and Steinheiser [1] highlighted how affective demands and organisational conditions interact with individual responses over time. Across the literature, cumulative processes were described through recurrence, layering, unresolved emotional burden, temporality and allostatic load.

#### 4.3.4. Effects on Nurses’ Health and Well-Being

The effects of cumulative occupational distress were described across psychological, emotional, professional, and organisational domains. Reported or conceptually linked outcomes included psychological distress, PTSD symptoms, burnout, compassion fatigue, moral distress, secondary traumatic stress, vicarious trauma, reduced resilience, fear for personal safety, avoidance of the workplace, reduced professional satisfaction, impaired well-being, and potential movement away from the profession. This pattern reinforces the conceptual gap identified in this review: nursing literature recognises the consequences of repeated occupational exposure, but does not consistently name or define the lower-intensity cumulative processes that may precede these outcomes.

## 5. Discussion

This scoping review examined how nursing literature conceptualises repeated occupationally distressing experiences and whether any terminology aligns with the emerging idea of micro-trauma. The absence of explicit micro-trauma terminology is significant because it highlights a gap not only in vocabulary, but in how nursing conceptualises cumulative precursor processes before end-point harms are recognised.

### 5.1. Terminology Fragmentation and Conceptual Overlap

The included evidence demonstrates the diverse terminology used to describe the sequelae of psychological trauma experienced by nurses. Cumulative trauma was the most relevant concept identified in the absence of micro-trauma terminology [16,23]. Other terms, including burnout, compassion fatigue, moral distress, vicarious trauma, secondary traumatic stress, and PTSD symptoms, functioned primarily as outcomes or related descriptors [1,5,15,25]. This pattern is consistent with the broader nursing literature, in which burnout, compassion fatigue, moral distress, psychological distress, and trauma-related symptoms are frequently examined as interrelated consequences of occupational stress rather than clearly differentiated cumulative processes [2,9,28]. This distinction matters because end-point terminology can obscure the cumulative process through which repeated exposure to grief, workplace aggression, moral constraint, resource insufficiency, emotional labour, and other distressing events becomes psychologically significant over time.

The inclusion of Bywood and McMillan [16] was undertaken with caution, recognising that the study aggregated data from multiple occupations and that nurses were not specifically analysed as a distinct group. Although this decision introduces a methodological limitation, the study provides indirect evidence that repeated exposure to work-related trauma, a phenomenon that cuts across emotionally demanding professions, may contribute to cumulative psychological harm. Its inclusion reflects the emerging and fragmented nature of the literature on cumulative occupational trauma, where similar concepts are described using variable terminology depending on the disciplinary field. Recognising this terminological fragmentation and the transversality of the underlying phenomenon is one contribution of this review to the nursing literature.

### 5.2. Conceptual Invisibility and the Problem of Naming Cumulative Precursor Processes

A key implication is that nursing appears to recognise cumulative occupational harm more readily at the point of consequence than at the point of emergence. Cumulative trauma provides language for repeated exposure, while end-point terms describe later or more visible consequences [16,23]. Without clearer terminology for the smaller repeated occupational experiences that contribute to cumulative trauma, exposure such as unresolved grief, moral constraint, repeated verbal aggression, resource insufficiency, errors, emotional labour, and repeated witnessing of suffering may remain normalised as ordinary nursing work [1,5,15,24,25]. This supports the need to examine the conceptual space before end-point harm occurs.

### 5.3. Theoretical Interpretation Through Stress, Coping, and Job Demands Frameworks

The transactional model of stress and coping [13] and the job demands–resources model [11,12] provide complementary ways to interpret the findings of this review. The transactional model explains why repeated occupational exposures do not produce uniform effects across nurses, as the meaning and impact of events such as death and dying, workplace aggression, moral constraint, resource insufficiency, or errors are shaped by individual appraisal, experience, perceived responsibility, controllability, and available support. The JD–R model extends this interpretation by locating cumulative occupational distress within the structure of work, where sustained emotional, cognitive, moral, physical, and organisational demands may contribute to a health-impairment pathway when adequate resources such as staffing, supervision, autonomy, debriefing, peer support, and organisational recognition are insufficient [11,12]. Read together, these frameworks help explain how repeated exposures may become meaningful at an individual level while also accumulating through organisational demands and resource limitations. However, neither framework provides specific terminology for the smaller, repeated, and often normalised occupational experiences that may occur before end-point harms are recognised. A future micro-trauma framework could therefore complement rather than replace existing stress and work-design theories by naming and organising the precursor space between exposure, appraisal, cumulative burden, and outcome. This supports the cautious position taken in this review: micro-trauma is not established in the nursing literature, but may offer a useful conceptual focus for future theory development and empirical testing.

### 5.4. Future Development of a Micro-Trauma Framework

Micro-trauma should therefore be positioned as a provisional area for future inquiry rather than an established nursing concept. The present review supports cumulative trauma as the closest evidence-aligned concept for understanding repeated occupational exposure in nursing. Future work may determine whether micro-trauma has a narrower role within cumulative trauma, for example, as a term for smaller, repeated, and often less visible occupational experiences that accumulate before end-point harms are recognised. This would require separate concept analysis, theory development, or empirical study.

A practical implication is that nursing research and workforce well-being initiatives should move upstream from end-point harm toward earlier recognition of cumulative exposure and cumulative burden. If future research can define and operationalise micro-trauma, it may support more precise assessment of recurring occupational exposures, clearer identification of cumulative precursor processes, and more targeted organisational responses. Until then, micro-trauma should be used cautiously as a possible framework for inquiry rather than an established explanation for nurses’ distress. This framing reinforces that responsibility for addressing cumulative occupational burden should not rest solely with individual nurses’ resilience, but also with the occupational conditions, repeated exposures, and organisational responses that shape nurses’ experience of work.

## 6. Study Limitations

This scoping review provides a broad synopsis of cumulative trauma in nursing; however, it is limited by the small number of included publications, heterogeneous terminology, and lack of consistent definitions across studies. Because the included literature did not explicitly use micro-trauma as a nursing concept, it could not be defined, validated, or established from the current evidence base. This review did not undertake a formal concept analysis either, and therefore did not identify defining attributes, model cases, antecedents, consequences, or empirical referents.

There was no consistent definition of what constituted an occupationally distressing or traumatic event or how repeated exposures were distinguished from cumulative trauma and end-point outcomes. One included source, Bywood and McMillan [16], was not nurse-specific and included healthcare workers, teachers, and social workers. This was retained as indirect cross-occupational evidence because it addressed repeated work-related trauma in comparable helping professions; however, its findings should not be interpreted as direct evidence about nurses.

The review was limited to English-language publications, and full texts were required to confirm eligibility and extract data. While every effort was made to access all identified manuscripts, records that could not be retrieved in full text could not be assessed, which may have introduced language and availability bias. The supplementary AI-assisted source identification pathway was also only partially reproducible because generative AI outputs may vary by tool version, date, and retrieval environment, even when identical prompts and standard platform settings are used. Searches were undertaken between March and May 2024, and the tools used have advanced substantially since that time, further limiting reproducibility. Google Scholar and grey literature searching were supplementary. Google Scholar screening was limited to the first three results pages, and grey literature searching was limited to .gov and .org domains. These limitations mean the findings should be interpreted as a map of current terminology and conceptual gaps rather than confirmation of micro-trauma as an established nursing concept.

## 7. Study Strengths and Implications

A strength of this review was the use of multiple complementary search approaches, including traditional database searching, citation and hand searching, Google Scholar, targeted grey literature searching, and AI-assisted source identification. Standard search methods involving large databases and established MeSH terms offer credibility; however, they may not always elicit findings for emerging or ambiguously defined concepts. In this review, AI-assisted source identification provided a contemporary supplementary means of identifying potentially relevant literature when traditional search methods did not yield included studies. This is consistent with the literature describing AI tools as potentially useful adjuncts for identifying hard-to-find literature while also requiring verification, transparency, and researcher judgement [20,22]. AI-identified results were used only to identify candidate records, and all AI-identified sources were checked against bibliographic databases, publisher information, and the review eligibility criteria before inclusion.

The main implication is that current end-point terminology may not fully capture the conceptual space before harm is recognised. Clearer terminology may support future research, nursing education, leadership training, and organisational policy by helping identify repeated occupational exposures and cumulative psychological burden earlier rather than after they manifest as burnout, compassion fatigue, moral distress, or PTSD symptoms.

## 8. Conclusions

This scoping review found that the nursing literature recognises the cumulative effects of repeated occupationally distressing experiences, but does not consistently name or define the lower-intensity cumulative processes that may preceded end-point harms. The term ‘micro-trauma’ was not identified as an established concept in the nursing literature. Instead, cumulative trauma, cumulative grief, workplace trauma, nurse-specific psychological trauma, emotional hazards, and end-point terms such as burnout, compassion fatigue, moral distress, secondary traumatic stress, vicarious trauma, and PTSD symptoms were used to describe related aspects of occupational harm.

These findings suggest an important conceptual space between routine occupational exposure and recognised end-point outcomes. Future research should develop and test a nursing-specific conceptual framework that can clarify whether micro-trauma is a useful and recognisable term for describing repeated, low-intensity, and often normalised occupational experiences that accumulate over time. Such work should avoid positioning occupational harm as an individual resilience issue alone and should account for the organisational, relational, moral, and resource-related conditions that shape nurses’ experience of work.

The findings have implications for nursing education, leadership development, workforce well-being, and organisational policy. Earlier recognition of cumulative exposure may support more proactive responses to staffing, supervision, debriefing, emotional labour, moral distress, workplace violence, supportive leadership, and trauma-informed organisational cultures. Expanding inquiry into the precursor space may help the profession identify not only the final straw, but also the accumulated burden of repeated occupational experiences over time.

## Figures and Tables

**Table 1:** Search strategy concepts organised using the SPICE framework.

SPICE Element	Operational Focus	Free-Text Keywords and Synonyms	Controlled Vocabulary and Subject Headings
Setting	Not restricted by setting	No setting-specific terms were applied.	Not applicable.
Perspective	Registered nurses and, in the broadened search, healthcare workers	nurs* OR ‘registered nurse*’ OR ‘healthcare worker*’ OR ‘health care worker*’ OR clinician*	Database-specific subject headings for nurses, nursing, and healthcare personnel were used where available.
Phenomenon of interest	Cumulative occupational distress and repeated low-intensity exposures	‘cumulative trauma’ OR ‘cumulative exposure’ OR ‘repeated exposure’ OR ‘occupational distress’ OR ‘workplace trauma’ OR ‘psychological stress’ OR ‘emotional labour’ OR ‘moral distress’	Medical subject headings (MeSH) and database-specific subject headings related to psychological stress, occupational stress, cumulative exposure, emotional labour, and moral distress were used where available.
Comparison	Not applicable	No comparison terms were applied.	Not applicable.
Evaluation	Nurses’ health, well-being and trauma-related outcomes	‘wellbeing’ OR ‘well-being’ OR burnout OR ‘compassion fatigue’ OR ‘moral distress’ OR ‘secondary traumatic stress’ OR ‘vicarious trauma’ OR PTSD OR ‘post-traumatic stress’ OR ‘post traumatic stress’	MeSH and database-specific subject headings related to post-traumatic stress disorder, burnout, occupational diseases, psychological stress and mental health outcomes were used where available.

Note. Controlled vocabulary differed across databases. Medical subject headings (MeSH) were used in MEDLINE, with equivalent subject headings applied in CINAHL and Emcare where available. Unexplained database abbreviations were removed for readability. Boolean operators were used to combine search concepts, with synonyms combined using OR and major concepts combined using AND. “*” denotes wildcard searching used.

**Table 2:** Source pathway for included publications.

Included Publication	Database Search: Nursing Terms	Broadened Healthcare Worker Search	AI-Assisted Source Identification	Google Scholar	Targeted Grey Literature Search
Davidson and Jackson [23]	No	No	Yes	No	No
Kapoor et al. [24]	No	No	Yes	Yes	No
Foli et al. [25]	No	No	Yes	No	No
Foli [4]	No	No	Yes	No	No
Boyle and Steinheiser [1]	No	No	No	Yes	No
Newman et al. [15]	No	No	No	Yes	No
Bywood and McMillan [16]	No	No	No	Yes	Yes

Note. ‘No’ indicates that the publication was not identified as a final included source through that search pathway. Google Scholar screening of the first three results pages identified six potentially relevant records for full-text retrieval and eligibility assessment. Not all candidate records met the final inclusion criteria. The initial nursing-specific database search and broadened healthcare worker database search retrieved records for screening, but did not yield eligible included publications. AI-assisted source identification, Google Scholar, and grey literature searching were supplementary identification strategies. All records identified through these pathways were independently verified and screened using the same eligibility criteria as database-derived records. Bywood and McMillan [16] was retained as indirect contextual/cross-occupational evidence and was not interpreted as nurse-specific evidence.

**Table 3:** Data extraction findings for included publications.

Author, Year, and Country	Purpose/Aim/Objective	Methodology	Population/Participants	Key Concept or Terminology	Major Relevant Findings	Relevance to Cumulative Occupational Distress
Davidson, P. & Jackson, C. [23]. United Kingdom	To deepen our understanding of trauma and its impact upon the mental health of nurses	Discursive paper	Nurses	Cumulative trauma	The presentation of long-term symptoms and maladaptive responses in nurses can be conceptualised as cumulative trauma.	Highlights how repeated exposures may contribute to cumulative psychological effects, aligning with the revised focus on cumulative trauma when micro-trauma was not identified.
Kapoor et al. [24] (USA)	To study the impact of the ritual of sacred pause on the attitudes and behaviours of the ICU physicians and nurses	Post-implementation survey analysis	ICU nurses and doctors	Cumulative grief	The implementation of a sacred pause was perceived to reduced cumulative grief and distress, improve resilience, promote teamwork and increase professional satisfaction.	Demonstrates the cumulative nature of repeated grief-related emotional stressors, supporting the focus on cumulative trauma rather than direct evidence of micro-trauma.
Bywood, P. & McMillan, J. [16] Australia	To identify and evaluate the effectiveness of prevention and intervention strategies addressing repeated exposure to work-related trauma, both direct and indirect	Systematic review	Occupational groups: healthcare workers (including nurses), teachers and social workers. This source was retained as indirect cross-occupational evidence and is not nurse-specific.	Cumulative trauma	Repeated exposure to trauma can trigger symptoms consistent with PTSD, compassion fatigue, secondary traumatic stress, vicarious trauma, and burnout.	Provides indirect contextual/cross-occupational evidence that repeated exposure to work-related trauma may contribute to long-term psychological harm. It is relevant to cumulative trauma but should not be interpreted as direct nurse-specific evidence.
Foli et al. [5]. USA	To describe nurses’ personal and professional trauma	Observational, mixed method, thematic analysis survey responses	Registered nurses	Nurse-specific psychological trauma	Identified nurses’ personal and professional trauma, including insufficient resource trauma, and differentiated avoidable and unavoidable trauma in nursing practice. The study also described outcomes and coping strategies associated with nurses’ trauma experiences.	Identifies specific nursing trauma exposures and cumulative burden, supporting the shift from absent micro-trauma terminology to cumulative trauma and nurse-specific psychological trauma.
Newman et al. [15]. Australia	To identify the key concepts related to the nature, extent, and impact of workplace trauma for forensic mental health nurses	Scoping review	Forensic mental health nurses	Cumulative trauma	Forensic mental health nurses were frequently exposed to workplace trauma and abuse. Reported impacts included psychological distress, fear for safety and workplace avoidance. Exposure to multiple forms of workplace trauma suggested that cumulative exposure over time may increase vulnerability to adverse personal and professional well-being outcomes.	Illustrates how repeated workplace trauma and abuse may contribute to cumulative distress, supporting cumulative trauma as the closest related concept.
Foli, K. [25]. USA	To present a new middle-range theory of nurses’ psychological trauma, theoretical statements, and outcomes of psychological trauma that are unique to nurses and their professional world	Discursive paper	Nurses	Nurse-specific psychological trauma	The paper presents a middle-range theory of nurses’ psychological trauma, outlining various types of traumas faced by nurses and their outcomes, with practical applications for understanding trauma in emergency department nurses during the COVID-19 pandemic.	Provides theoretical grounding for nurse-specific psychological trauma and cumulative burden without establishing microtrauma as a named concept.
Boyle, D. A. & Steinheiser, M. M. [1]. USA	Review the sequelae of emotional hazards and provide recommendations for organisational and practice setting changes to promote nurse well-being and identify opportunities for future innovation and research	Review	Nurses	Cumulative emotional hazards	The paper highlights affective demands inherent in nursing work and identifies organisational and practice-level emotional hazards that require intervention to support nurse well-being.	Emphasises that routine nursing work can carry cumulative emotional demands, informing the review’s focus on cumulative trauma and cumulative occupational burden.

Note. This table summarises the included publications and identifies their relevance to cumulative trauma after micro-trauma was not identified as an explicit term in the nursing literature.

**Table 4:** Cumulative trauma and cumulative exposure terminology identified in the included literature.

Cumulative Trauma-Related Term	How the Term Is Used in the Literature	Exposure, Process, or Outcome Emphasis	Relevance to the Review Question
Cumulative trauma	Describes effects of repeated or prolonged exposure to direct and indirect occupational trauma, including workplace violence, emotionally distressing events and cumulative professional stressors.	Emphasises repetition, layering and cumulative process, often linked to later psychological outcomes.	Closest adjacent concept; captures accumulation over time but does not differentiate smaller, lower-intensity exposures that might align with micro-trauma.
Cumulative grief	Used in relation to repeated exposure to death, dying and loss, particularly in intensive care and palliative care contexts.	Highlights repeated emotional exposure, unresolved grief and temporality as cumulative processes.	Illustrates how routine emotionally significant events may accumulate but remains specific to grief-related exposure.
Workplace trauma	Refers to exposure to workplace violence, aggression, abuse, threats or unsafe environments.	Primarily emphasises occupational exposure, with psychological distress, fear, avoidance and PTSD symptoms as outcomes.	Relevant where repeated lower-intensity aggression or threat contributes to cumulative distress, though the term often includes more overt traumatic events.
Nurse-specific psychological trauma	Describes trauma arising from the professional world of nursing, including system-induced trauma, vicarious trauma, insufficient resource trauma and second-victim trauma.	Emphasises nursing-specific exposures, cumulative burden and effects on well-being and professional functioning.	Provides theoretical grounding for nursing-specific trauma but does not explicitly name micro-trauma as a distinct construct.

Note. This table summarises cumulative trauma and cumulative exposure terminology identified in the included literature. End-point terms are discussed as outcomes or sequelae where relevant, but are not treated as mapped concepts in this review.

**Table 5:** Study-level mapping of accumulation across included publications.

Included Paper	Concept Used	How Accumulation Is Addressed	Relevant Accumulation Dimensions	End-Point Outcomes Described or Implied
Davidson and Jackson [23]	Cumulative trauma	Describes trauma as delayed and cumulative, with nurses’ personal and professional experiences layering over time and contributing to long-term symptoms and maladaptive responses.	Repetition; temporality; delayed response; layered exposure; personal and professional trauma history.	Long-term psychological symptoms, maladaptive coping and delayed post-traumatic stress responses.
Kapoor et al. [24]	Cumulative grief	Frames repeated exposure to patient death and dying in intensive care as cumulative grief requiring ritualised acknowledgement (e.g., sacred pause).	Repeated death exposure; grief; unresolved emotional burden; temporality; ritualised processing.	Distress, burnout risk, grief-related burden, resilience and professional satisfaction.
Bywood and McMillan [16]	Cumulative exposure to work-related trauma	Synthesises evidence across occupational groups on repeated direct and indirect trauma exposure at work. Treated as indirect evidence because nurses were not analysed separately.	Repeated exposure; direct and indirect trauma; cross-occupational cumulative burden; prevention and intervention focus; indirect relevance to nursing.	PTSD symptoms, compassion fatigue, secondary traumatic stress, vicarious trauma and burnout.
Foli et al. [25]	Nurses’ psychological trauma	Identifies multiple trauma types in nursing, including system-induced trauma, second-victim trauma, vicarious trauma, workplace violence, disaster trauma and insufficient resource trauma.	Multiple trauma types; organisational exposure; errors or witnessed errors; insufficient resources; cumulative burden; allostatic load.	Psychological distress, coping burden, impaired well-being and professional functioning.
Newman et al. [15]	Workplace trauma and cumulative exposure	Describes repeated exposure to workplace violence and abuse in forensic mental health nursing, including physical violence, verbal abuse, sexual violence, self-harm events and threats to safety.	Violence; repeated exposure; cumulative workplace trauma; fear for safety; workplace avoidance; temporality.	Psychological distress, PTSD symptoms, burnout, fear for safety, reduced well-being and workplace avoidance.
Foli [5]	Middle-range theory of nurses’ psychological trauma	Theorises nurses’ psychological trauma as arising from multiple sources within the professional world of nursing, linking repeated stress and trauma exposure to cumulative burden and allostatic load.	Theory development; cumulative burden; allostatic load; system-induced trauma; vicarious trauma; insufficient resource trauma; second-victim trauma.	Declining physical and mental health, impaired professional functioning, professional distress and potential effects on workforce retention.
Boyle and Steinheiser [1]	Emotional hazards of nurses’ work	Addresses repeated affective demands inherent in nursing work and describes organisational and practice-level emotional hazards associated with adverse sequelae.	Emotional labour; organisational exposure; repeated affective demands; moral distress; compassion fatigue; burnout; intervention planning.	Burnout, compassion fatigue, moral distress, reduced well-being and need for organisational intervention.

Note. This table maps how included studies address accumulation through repetition, temporality, unresolved exposure, grief, violence, allostatic load, and end-point outcomes. It does not imply that the included studies explicitly used or validated the term ‘micro-trauma’.

## Data Availability

No new data were created or analysed in this study. Data sharing is not applicable to this article.

## Supplementary Materials

The following supporting information can be downloaded at https://www.mdpi.com/article/10.3390/nursrep16090299/s1. Table S1: Preferred Reporting Items for Systematic reviews and Meta-Analyses extension for Scoping Reviews (PRISMA-ScR) Checklist.

## Public Involvement Statement

No public involvement in any aspect of this research.

## Guidelines and Standards Statement

This scoping review was conducted and reported with the PRISMA Extension for Scoping Reviews (PRISMA-ScR) guidelines [18]. The completed PRISMA-ScR checklist is provided in the Supplementary Materials (Table S1).

## Use of Artificial Intelligence

During the preparation of this manuscript, the authors used generative artificial intelligence tools (Perplexity (https://www.perplexity.ai/), Microsoft Copilot (https://copilot.microsoft.com/), and Elicit (https://elicit.com/)) to support supplementary source identification. AI tools were used to identify candidate sources only. All outputs were reviewed, verified against bibliographic or publisher information, screened against eligibility criteria, and interpreted by the authors, who take full responsibility for the content of this publication.

## Abbreviations

The following abbreviations are used in this manuscript.

AI	Artificial Intelligence
CINAHL	Cumulative Index to Nursing and Allied Health Literature
Emcare	Elsevier Nursing and Allied Health Database
ICU	Intensive Care Unit
JD-R	Job Demands-Resources
JBI	Joanna Briggs Institute
MeSH	Medical Subject Headings
MEDLINE	Medical Literature Analysis and Retrieval System Online
*n*	number
NSW	New South Wales
PRISMA-ScR	Preferred Reporting Items for Systematic Reviews and Meta-Analyses Extension for Scoping Reviews
PTSD	Post-Traumatic Stress Disorder
SPICE	Setting, Perspective, Intervention, Comparison, Evaluation
USA	United States of America
WHO	World Health Organization
.gov	Government domain
.org	Organisation domain
